# UV Laser-Induced, Time-Resolved Transcriptome Responses of *Saccharomyces cerevisiae*

**DOI:** 10.1534/g3.119.400291

**Published:** 2019-06-18

**Authors:** Melinda Hauser, Paul E. Abraham, Lorenz Barcelona, Jeffrey M. Becker

**Affiliations:** * Department of Microbiology, University of Tennessee, Knoxville, TN 37996 and; † Chemical Sciences Division, Oak Ridge National Laboratory, Oak Ridge, TN 37831

**Keywords:** yeast, gene expression, RNAseq, UV-laser-induced response

## Abstract

We determined the effect on gene transcription of laser-mediated, long-wavelength UV-irradiation of *Saccharomyces cerevisiae* by RNAseq analysis at times T15, T30, and T60 min after recovery in growth medium. Laser-irradiated cells were viable, and the transcriptional response was transient, with over 400 genes differentially expressed at T15 or T30, returning to basal level transcription by T60. Identification of transcripts exhibiting enhanced differential expression that were unique to UV laser-irradiation were identified by imposing a stringent significance cut-off (*P* < 0.05, log_2_ difference >2) then filtering out genes known as environmental stress response (ESR) genes. Using these rigorous criteria, 56 genes were differentially expressed at T15; at T30 differential expression was observed for 57 genes, some of which persisted from T15. Among the highly up-regulated genes were those supporting amino acid metabolic processes sulfur amino acids, methionine, aspartate, cysteine, serine), sulfur regulation (hydrogen sulfite metabolic processes, sulfate assimilation, sulfate reduction), proteasome components, amino acid transporters, and the iron regulon. At T30, the expression profile shifted to expression of transcripts related to catabolic processes (oxidoreductase activity, peptidase activity). Transcripts common to both T15 and T30 suggested an up-regulation of catabolic events, including UV damage response genes, and protein catabolism via proteasome and peptidase activity. Specific genes encoding tRNAs were among the down-regulated genes adding to the suggestion that control of protein biosynthesis was a major response to long-wave UV laser irradiation. These transcriptional responses highlight the remarkable ability of the yeast cell to respond to a UV-induced environmental insult.

Absorption of ultraviolet (UV) radiation by living cells has the potential to damage nucleic acids and proteins either by direct energy absorption or indirectly through the generation of free radicals or singlet oxygen species (Basu 2018). It is well-documented in humans that excessive exposure to UV radiation results in a variety of skin cancers and damage to the lens of the eye (Modenese *et al.* 2018). In yeast, exposure to short-wave UV radiation results in damage to DNA, stimulating mitotic recombination-based repair which is largely completed during the first cell cycle after irradiation (Yin and Petes 2013).

Most of the research on UV carcinogenesis has concentrated on short-wavelength radiation (280-315 nm), although radiation of long-wavelengths also may be carcinogenic. Whereas short-wavelength UV is absorbed by DNA, long-wavelength radiation is toxic by indirect mechanisms in which reactive oxygen species are generated after the radiation is absorbed by cellular molecules (de Laat *et al.* 1996).

Our impetus for using laser-mediated irradiation is to cross-link receptor-ligand and receptor-intracellular protein interactions in live yeast in a time-resolved manner. UV light is routinely used to cross-link photoactivatable groups to proteins of interest (Pham *et al.* 2013). Generally, the protocol involves incubation for tens of minutes under UV bulbs emitting light at 360 nm. A laser provides high power radiation using monochromatic, collimated light. These optical properties lead to high radiance (emitter power per unit area cross-section of the beam) enabling focusing of the high power to a small area with high local fluence rates.

In a previous study using UV-laser irradiation, we induced a rapid cross-linking of a peptide labeled with photoactivatable amino acid analog *p*-benzoyl-L-phenylalanine (Bpa) into a binding pocket of bovine serum albumin (Hauser *et al.* 2018). This protocol allowed cross-linking of the ligand to the substrate in a matter of seconds, in contrast to longer exposure required under a conventional UV lamp. This established the feasibility of using laser-induced cross-linking to capture protein-protein interactions in a live yeast cell in a matter of seconds in a time-resolved manner.

To extend this methodology to the irradiation of a whole cell, it was necessary to determine whether a short burst of long-wavelength UV energy impacted cell viability or affected gene expression that would influence the analysis of whole cell protein cross-linking. Depending on the experimental conditions, changes in gene expression could influence the results of cross-linking studies by altering expression of genes encoding target proteins. In this correspondence, we conducted an examination of early changes in the yeast transcriptome subsequent to a short burst of laser energy. Within 15 min of inoculating laser-irradiated cells into fresh medium, it was determined that there was a change in the transcriptional profile of hundreds of genes, including a subset of environmental stress response (ESR) genes (Gasch *et al.* 2000). The change was transient, with differential expression persisting for 30 min before returning to near baseline levels by 60 min underline the rapid ability of the yeast cell to respond to a UV-induced environmental insult. The most highly expressed genes appeared to be those involved in protein synthesis and degradation and in oxidative repair mechanisms.

## Materials and Methods

### Strain and growth conditions

Experiments were conducted using *Saccharomyces cerevisiae* strain BY4741 (*MATa his3Δ1 leu2Δ0 met15Δ0 ura3Δ0*), a direct descendant of *Saccharomyces cerevisiae* S288C (see Reagent Table). Apart from its auxotrophies, BY4741 is essentially identical to S288C, the strain used for the systematic yeast genome sequencing project (Goffeau *et al.* 1996; Engel *et al.* 2014) which serves as the reference genome for this study. BY4741 cultures were maintained on YEPD solid medium (1% yeast extract, 2% peptone, 2% glucose, 2% agar) incubated at 30°. For liquid cultures, strains were grown in an orbital shaking incubator (30°, 150 RPM) in YEPD without agar.

### Laser irradiation

The Explore One XP 355-1 UV laser (Spectra-Physics, Santa Clara CA) controlled by L-Win, a LabView-based graphical user interface, was used to irradiate samples. The software indicated that the energy per pulse at a pulse repetition frequency was 50 kHz and laser output set at 75% maximum amperage was 36.7 × 10^−6^ J/pulse. Irradiating the sample under these conditions for 30 sec results in the delivery of approximately 55 J. The beam was directed through a plano-concave lens (f 75.0 mm, Ø1” UV Fused Silica, Thorlabs, Newton, NJ) to expand the diameter to approximate that of the diameter of the sample tube (10 mm), and the beam was reflected off a fused silica UV-laser mirror (Thorlabs, Newton, NJ) down into the tube. To begin the irradiation, the tube containing the sample was placed into a benchtop freezer block, chilled to -20°, the block positioned such that the aperture of the tube was in alignment with the beam, and the samples were irradiated. Upon completion of the 30s irradiation interval, the tubes were stored on ice until used as described below.

### Measurement of cell growth

Three biological replicates of BY4741 (40 mL cultures) were grown overnight and each were processed identically. Mid-log phase cells were harvested by centrifugation and washed twice with 25 mL sterile phosphate buffered saline (PBS: 10 mM Na_2_HPO_4_, 1.5 mM KH_2_PO4, 3 mM KCl, 150 mM NaCl, pH 7.4) and finally re-suspended in 3 mL PBS. Two 0.5 mL samples were placed into 2 mL flat bottom Eppendorf tubes and were kept on ice. One of the two samples was laser-irradiated for 30s as specified above, the second was not irradiated and served as a control. Following laser treatment, control and laser-irradiated cells were counted and diluted to a concentration of 2 × 10^6^ cells/ml in YEPD. Four technical replicates (200 μL) of each of the three biological replicates for both control and laser treated samples were added to a 96-well plate. The plate was incubated in the spectrophotometer (BioTek PowerWave 340, Winooski, VT) at 30° with shaking. The A_600_ was measured every 30 min to monitor the growth of the cells until stationary phase was reached.

### Trypan blue exclusion

A small portion of the laser-irradiated and control cells prepared for the growth curve (above) was diluted to 4 × 10^8^ cell/mL (100 µL total volume), mixed with 100μL of 0.4% Trypan blue with 0.85% NaCl for microscopic examination. In addition, a non-viable control was prepared by heating cells (100 µl, 4 × 10^8^ cells/mL) to 100° for 10 min prior to trypan blue staining. The slides were imaged on a Keyence BZ-X710 Microscope (Itasca, IL) set for bright field, and images were collected using the 40x objective. The color, white balance and exposure were maintained across all images. The percent viability was determined for laser treated and control cells across four different fields. Data reported are the average of the determination across the three biological replicates.

### Post-Irradiation time course for RNASeq

The process described below was repeated in triplicate for a total of three biological replicates. An overnight culture of BY4741 was used to inoculate a 50mL culture of YEPD to a density of 2 × 10^7^ cells/mL which was then grown for an additional 5 hr until mid-log phase. The cells were harvested by centrifugation, washed, re-suspended in 1 mL PBS, chilled on ice and counted. The cells were adjusted to a final volume of 6mL in PBS at a concentration of 5 × 10^8^ cells/mL. The 6 mL of cells were split into two 3 mL portions and held on ice. The first was left on ice while the second was further divided into 6 × 0.5 mL cultures in 2.0 mL flat bottom Eppendorf tubes and laser irradiated as described above. Upon completion of laser irradiation, the 6 × 0.5 mL portions were combined back into a single 3 mL sample. Laser and non-laser treated groups (3 mL) were added to 100 mL YEPD pre-warmed to 30° (final density of 1.5 × 10^7^ cells/mL). Immediately upon inoculation, a 21 mL sample of the culture was removed and labeled as Time 0 (zero incubation time in the YEPD post-inoculation) and the remaining 82 mL of culture was placed into an orbital shaking incubator. One mL of the 21 mL sample was used to measure optical density. The remaining 20 mL were harvested by centrifugation at 4°, washed with ice cold PBS, harvested, re-suspended in 1 mL of ice-cold PBS, and transferred to a 2 mL Eppendorf tube. The cells were then harvested again, the PBS was removed, and the cell pellet was promptly snap frozen in dry ice-ethanol. The entire harvest and freezing process took approximately 6 min. Subsequent 21 mL samples were removed at 15 (T15), 30 (T30), and 60 (T60) minutes post-inoculation and processed as described for the Time 0 sample. The snap frozen cells were stored at -80° until processed for RNA extraction.

### RNA isolation and integrity check

The frozen cell pellets were processed for RNA extraction as previously described (Fozo *et al.* 2010). Briefly, the protocol entails bead-beating in acid phenol/chloroform, followed by a second extraction in hot acid phenol/chloroform, followed by repeated extractions with phenol/chloroform until the aqueous/organic interface was clean. The RNA was then precipitated in ethanol, dissolved in RNAse-free water and the concentration determined spectrophotometrically.

RNA integrity was verified by gel electrophoresis to ensure that two distinct bands corresponding to 18s and 26s rRNA subunits with minimal to no smearing was present. The samples were run on a 2100 Bioanalyzer using a High Sensitivity Total RNA Analysis Chip (Agilent, Santa Clara, CA) to assess RNA purity and concentration. The samples were run using the Plant RNA Nano Assay because the yeast 26S is closer to the plant 25S than the eukaryotic 28S rRNA and is specified by the manufacturer to be the most appropriate analysis for yeast RNA. The 2100 Bioanalyzer outputs a RIN score (RNA Integrity Number) which is a meter of RNA quality where a score above 6 indicates high quality RNA.

### Library preparation and sequencing

Library preparation and sequencing was conducted by the University of Tennessee Genomics Core. The Tru-Seq Stranded mRNA kit (Illumina, San Diego, CA) was used on 2 µg of total RNA to enrich mRNA, create cDNA, and prepare the library for sequencing. Library quality was verified using the DNA 1000 Assay Chip on the 2100 Bioanalyzer (Agilent, Santa Clara, CA). This assay detects the presence of artifacts from inefficient ligation, poor DNA quality, primer dimers, and PCR artifacts from over-amplification, but is primarily used to assess concentration. Sequencing was performed using the MiSeq 2x75bp paired end sequencing kit (Illumina, San Diego, CA) which produces 44-50 million paired-end reads.

### Processing of RNAseq data

The sequencing reads were analyzed using Lasergene SeqMan NGen version # 14.1.0(115) software (DNASTAR, Inc, Madison, WI). The assembly workflow was set to Transcriptome/RNAseq and the assembly type was set to Reference based assembly with the *Saccharomyces cerevisiae* S288C (NCBI BioProject: PRJNA43747) genome as the reference genome. The remaining settings were left at default values. Differential gene expression analysis was then performed in DNASTAR Lasergene ArrayStar version # 14.1.0 build 172. The false discovery rate (FDR) (Benjamini and Hochberg 1995) method is the default P-value adjustment method in Arraystar and it was used to determine percent confidence of differentially expressed genes (DEGs).

### Clustering and gene ontology enrichment

For the subset of transcripts that were differentially expressed between four time points (T0, T15, T30, and T60) (Table S1), two-way hierarchical clustering (Euclidian) was performed on the Log2 fold changes from each pairwise comparison using the Perseus software (Tyanova *et al.* 2016). Using ClueGO (Bindea *et al.* 2009), clusters were individually tested for over-represented GO biological process using a right-sided hypergeometric enrichment test at medium network specificity selection. P-value correction was performed using the Holm-Bonferroni step-down method (Holm 1979). There was a minimum of 3 and a maximum of 8 selected GO tree levels, while each cluster was set to include a minimum of between 3% and 4% of genes associated with each term. The GO terms at adjusted *P* < 0.05 were considered significantly enriched (Table S1).

### Bray-Curtis clustering, NMDs, ANOSIM, GO, and VOLCANO plots

For DEGs identified at each time point, Bray-Curtis clustering was done in PRIMER version 7.0.12 (Primer-E, Quest Research Unlimited, Auckland, NZ). The RPKM values from each replicate for each sample was square root transformed and then measured for Bray-Curtis similarity. Non-metric multidimensional scaling (nMDS) was then performed on the resemblance data. A 1-Way Analysis of Similarities (ANOSIM) for treatment and time was performed on the resemblance data to measure variation between the samples and similarity between the replicates within different samples. GO enrichment analysis was performed using GO Slim Mapper from the Saccharomyces Genome Database (Cherry *et al.* 2012). Volcano plots (Li 2012) were used to show changes in the data set of replicates. This plot is a representation of significance *vs.* fold-change on the y and x axes, respectively, of individually expressed genes.

### Data availability

Illumina MiSeq FASTQ files of the paired-end reads for each sample and the associated processed data file are available online at NCBI’s Gene Expression Omnibus here [GEO Submission (GSE129319)]. Supplementary material cited throughout the manuscript (Figure S1, Tables S1-S4, and File S1) and Reagent Table have been deposited at FigShare: https://doi.org/10.25387/g3.8047727.

## Results and Discussion

### Effect of laser irradiation on cell viability

**Figure 1:**
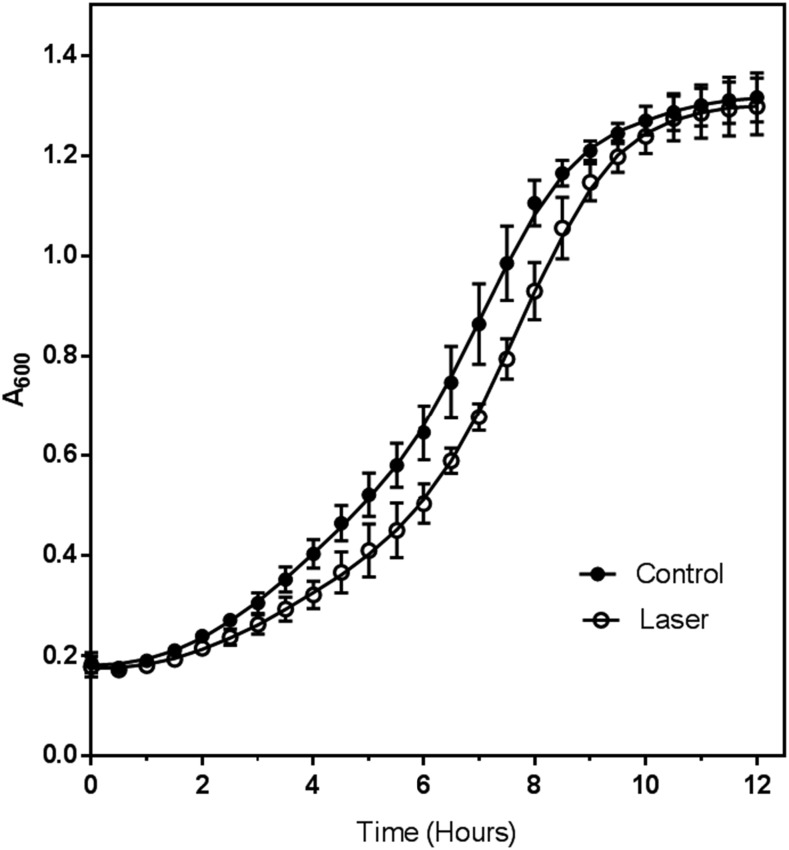
Growth of control (**●**) and laser irradiated (○) cells upon transfer into growth medium. For each condition, the optical density at 600 nm (A_600_) was determined for three independent biological replicates. Each biological replicate was measured in quadruplicate and the average value for the biological and technical replicates plotted as a function of time.

Prior to interrogating the transcriptome of laser irradiated cells, the conditions used in this experiment were assessed for their impact on cell viability via both trypan blue exclusion and growth assays. Trypan blue is a vital stain which is excluded from live cells. Laser irradiated and non-irradiated cells were stained with trypan blue, and images were collected and scored for the presence of nonviable (blue-stained) cells (Fig. S1). For comparison, heat-killed cells were included as a control. As expected, 100% of the heat killed cells were stained (non-viable), while less than 1% of the non-irradiated cells were stained. The laser-treated cell population had 3–4% non-viable cells. These data suggested that laser irradiation did not grossly damage cells. To verify that the irradiated cells were still able to divide, cells (irradiated and non-irradiated) were inoculated into fresh medium and the A_600_ measured every thirty minutes until stationary phase was reached. The growth curves (Figure 1) indicate that following a lag phase, the doubling time of both the control (140 ± 3 min) and laser-treated cells (137 ± 14 min) were not significantly different (*P* > 0.05, two-tailed T-test). Under these conditions DNA damage was expected to be minimal in contrast to radiation at 254nm that is commonly used for radiation-induced DNA damage in yeast (Guintini *et al.* 2017; Li *et al.* 2018). The strong laser output we used allowed for a much shorter exposure time (30 sec) and a longer wavelength (355nm) that corresponded to the conditions used for our previous protein-peptide cross-linking experiments (Hauser *et al.* 2018).

The delay in growth initiation indicated by the longer lag phase of irradiated cell was the result of cell metabolic responses to UV that are described in the RNAseq experiments presented below in this report. The expected doubling time for yeast grown in complete medium is approximately 90 min (Sherman 2002); the increased doubling time measured in this experiment is due probably to reduced aeration of the cells while growing in a plate reader. Coupled with the trypan blue staining, the growth assay indicated that the yeast cells were mostly viable after laser treatment and therefore further interrogated by transcriptomic analysis.

### Laser irradiation induces differential gene expression

**Figure 2:**
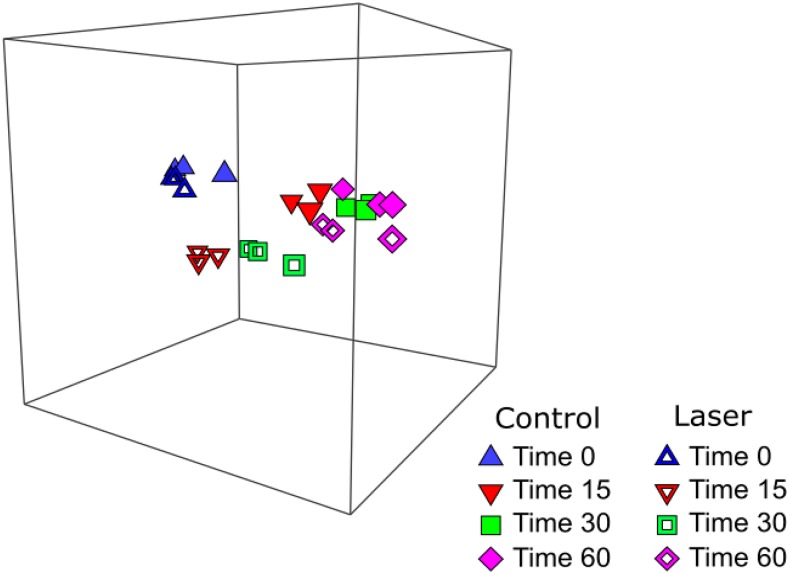
Three-dimensional non-metric multidimensional scaling (3D-NMDS) plot of control (solid symbols) and laser treated (open symbols) samples collected at 0, 15, 30, and 60 min post-inoculation into fresh medium. Three independent biological replicates are plotted for each condition at each time point. The distance between points represents the differences between the samples. The low stress value (3D stress = 0.02) of the ordination indicates a good fit. An additional view of a rotating model is available (File S1).

To assess gene expression post-laser irradiation, total RNA was extracted from both control and 30s laser-irradiated yeast cells grown for various times (0, 15, 30 and 60 min) after irradiation and inoculation into fresh medium. The RNA was processed to isolate mRNA, which was then subjected to RNAseq analysis. To examine global changes in gene expression in response to laser irradiation, a three-dimensional Bray-Curtis based non-metric multidimensional scaling (nMDS) plot (File S1) was created using the RPKM values for every gene from every sample. A snapshot of the three-dimensional image is shown in Figure 2. The nMDS plot suggested similar gene expression when comparing control (closed) and laser-treated (open) samples at T0 (blue triangles) and T60 (pink diamonds), evidenced by the clustering of the three biological replicate points for each treatment groups. In contrast, T15 (red inverted triangles) and T30 (green squares) samples exhibited greater differentiation between control (open symbols) and laser (closed symbols) treatment groups in comparison to the differences at 0 and 60 min. The stress value, which represents the difference between distance in the reduced dimension compared to the complete multidimensional space, is low (3D Stress: 0.02) for the nMDS plot thus verifying that this is an accurate, low dimension representation of the observed distances among the samples. Variation in treatments and composition among replicates was further evaluated by ANOSIM to determine the effect of treatment (control v. laser) and time (0, 15, 30 or 60 min) on the sample sets (Table S2). The R statistic signifies no difference between treatment groups when the value is 0. When R approaches or is equal to 1, the difference between treatments being compared is significantly greater than the difference among the replicates within the treatments. At T0 and at T60, control *vs.* laser-treated samples exhibit low R values of -0.037 and 0.037, respectively, suggesting little differences between the treatment groups as reflected in the nMDS plot (Figure 2). In contrast, laser-treated *vs.* control samples at T15 and T30 display high R values of 0.963 and 0.704, respectively, suggesting significant differences in gene expression for laser-treated *vs.* control groups at these time points. The nMDS plot indicates that changes in the transcriptome in response to laser irradiation are transient and have returned to baseline levels by 60 min post transfer to growth medium. Given the shift to normal growth and strong similarities between the control samples and the samples measured 60 min post-transfer, these data suggest that the cells have repaired any damage induced by the UV irradiation by 60 min. To further investigate this finding, we characterized the transient transcriptional responses, which is detailed below.

### DEGs in response to laser irradiation is distinct from stress response

**Figure 3:**
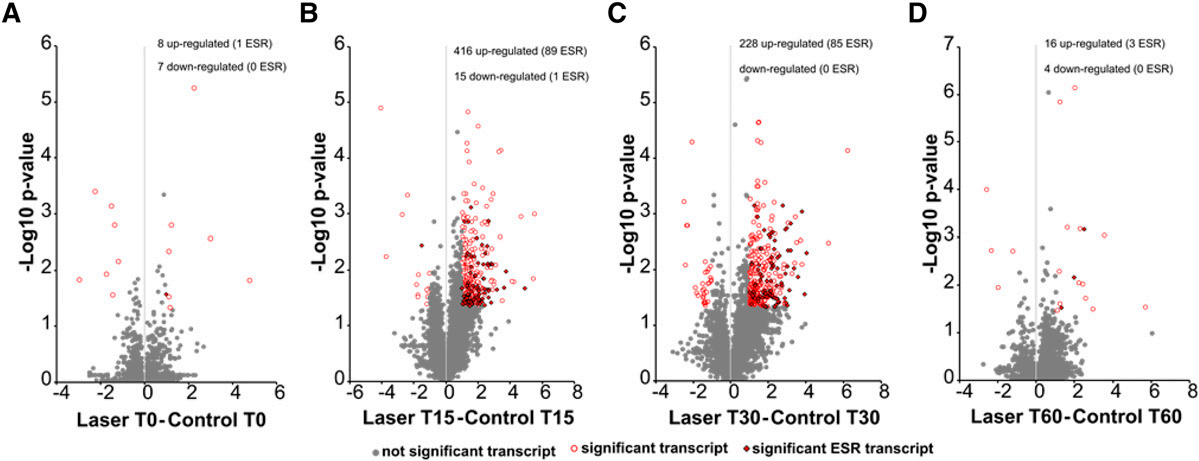
Volcano plot distribution of differential transcripts. ANOVA (F-test) was used to compare the means of gene expression from the triplicate RPKM values for each gene in the presence and absence of laser treatment to determine a P-value. The –Log10 p-value was plotted as a function of differential expression (Log2 difference) to generate the volcano plot. For each pairwise comparison (laser v. control) at (a) time 0, (b) time 15, (c) time 30 and (d) time 60 min post inoculation into fresh medium, transcripts with a p-value < 0.05 and a Log2 difference > 1 were considered to be significantly different and are highlighted in red. Significant transcripts which are part of the environmental stress response (*ESR* transcript) are indicated by solid red diamonds. Transcripts which exhibit significant differential expression unique to laser irradiation are indicated by open red circles. Table S1 lists all of these DEGs.

Volcano plots (Figure 3A-D, also shown in Table S1) of significance *vs.* fold change over time were constructed to visualize the differentially expressed genes (DEGs). Transcripts considered significantly different (p-value <0.05, log2 difference >1) are highlighted in red and specific genes meeting these criteria are listed in Table S1. A specific subset of approximately 900 genes respond universally to a wide range of environmental stress in yeast (Gasch *et al.* 2000). These environmental stress response (ESR) genes are indicated by a small diamond on the “circle” in the volcano plots (Figure 3) to define the overlap between the general stress response and DEGs in response to laser irradiation. At time T0 (Figure 3A) there was little difference in gene expression comparing laser-treated and control groups, and only 1 of the 8 up-regulated DEGs was identified as an ESR gene. In contrast, samples collected at T15 (Figure 3B) exhibited up-regulation of 416 genes, including 80 ESR genes, and down-regulation of 15 genes none of which are ESR genes. At T30 (Figure 3C) differential gene expression was reduced to 228 up-regulated genes, but the number of ESR genes within this population (89 ESR genes) was similar to those observed at T15. By T60 (Figure 3D) differential expression had returned to levels similar to those observed for T0. The data indicate that differential expression in response to laser irradiation under the conditions used in this experiment is rapid and transient. There is some overlap with ESR genes, but stress response does not account for all DEGs, and not all ESR genes responded to the laser treatment.

**Figure 4:**
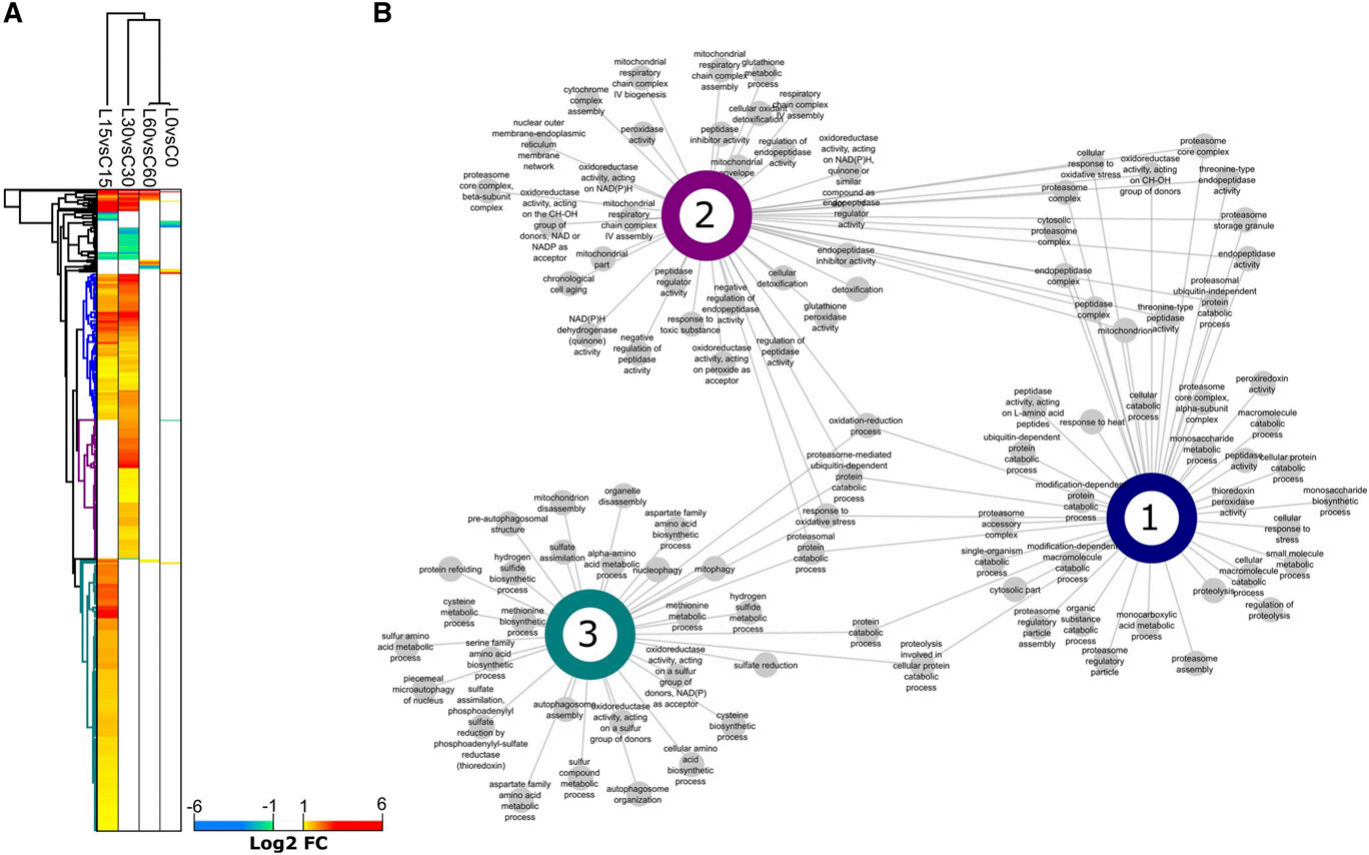
Differential transcript abundance behaviors and network clustering of gene ontology (GO) functional intersections between the most prevalent differential gene expression patterns. (a) Two-way hierarchical clustering (Euclidian) of Log2 fold changes for each pairwise comparison was performed in the Perseus software for on only the differentially abundant transcripts observed. Non-significant transcript differences were replaced by a zero value. Here, we highlight the three major discrete clusters representing those transcripts that were upregulated in time points 15 and 30 min (blue), and those that were up-regulated in only the 15 min (cyan) and 30 min time points (purple). (b) For each of the three clusters, we performed GO enrichment to identify the functional responses associated with each differential expression pattern. The node connectivity of significantly enriched GO terms is represented for each cluster as well as between clusters, which is conceptually like a Venn diagram. The GO network highlights the number of enriched terms for each cluster as well as the functional intersection, for example GO terms shared between all hierarchical cluster (1-3) represent a sustained functional response. The GO terms at adjusted *P* < 0.05 were considered significantly enriched and are highlighted.

The DEGs were further analyzed by two-way hierarchical clustering (Figure 4A) to identify transcripts that were upregulated at T15, T30 or at both T15 and T30. Gene ontology (GO) enrichment was performed on each transcript cluster to identify functional responses and their connectivity within and between clusters (Figure 4B). At T15 (Figure 4B, Cluster 3) amino acid metabolic processes (sulfur amino acids, methionine, aspartate, cysteine, serine) are highly represented in addition to sulfur regulation (hydrogen sulfite metabolic processes, sulfate assimilation, sulfate reduction). At T30 (Figure 4B, Cluster 2), the expression profile shifts to expression of transcripts related to catabolic processes (oxidoreductase activity, peptidase activity). Transcripts common to both T15 and T30 time points (Figure 4B, Cluster 1) suggest an upregulation of catabolic events, including protein catabolism via proteasome and peptidase activity. Expression of genes involved in DNA repair was minimal at T15 or T30 indicating that the 355nm UV-irradiation used in this experiment did not cause extensive DNA damage, although it has been shown previously by others that transcriptional response of *S. cerevisiae* to short-wavelength UV radiation that damages DNA may not directly identify genes that protect against UV radiation (Birrell *et al.* 2002). The irradiation conditions used in this experiment appeared to trigger responses dealing with repair of oxidative damage to proteins and cellular components. Twenty-two genes involved in oxidative repair were highly up-regulated at T30 (Table S4).

### Iron regulon transcripts exhibit differential expression in response to UV laser irradiation

The top ten non-ESR genes, exclusive of genes encoding tRNAs (see below), exhibiting the highest level of differential expression at T15, T30, and T60 are presented in Table 1 and Table S3. At T15, the gene *LSO1* (*l*ate-annotated *s*mall *o*pen reading frame) exhibited ∼45-fold change (FC) (Log_2_ FC = 5.5) increase in expression, rising to ∼73-fold (Log_2_ FC = 6.2) at T30, representing the most highly modulated gene at these time points. By T60, expression had returned to basal level and was not significantly different (*P* > 0.05) from the control levels. *LSO1*, a member of the Aft1p/Aft2p-regulated iron regulon encodes a 93 amino acid protein that is involved in the cellular response to iron deprivation (An *et al.* 2015). Its role in the iron regulon prompted a further interrogation of the dataset for other genes transcriptionally activated by transcription factors Aft1/Aft2 in response to iron deficiency (Martínez-Pastor *et al.* 2017; An *et al.* 2015) which identified 12 additional differentially expressed (Log_2_ FC ≥ 1) transcripts (Table 2). All of these genes, including metalloreductases, ion transporters, an iron-recycling heme oxygenase, and a copper-transporting ATPase, were differentially up-regulated at T15. Six transcripts (*LSO1*, *HMX1*, *TIS11*, *CCC2*, *FRE3* and *FIT2*) were differentially expressed at both T15 and T30, while only *FIT2*, an iron siderophore transporter, was up-regulated at all three time points. None of the iron regulon transcripts identified in this study as modulated in response to laser irradiation were identified as part of the ESR gene dataset (Gasch *et al.* 2000) suggesting that this response is distinct from a general cellular stress response.

**Table 1:** Top 10 differentially expressed, non-ESR transcripts upregulated in response to laser irradiation at time T15, T30 and T60 min post-transfer to growth medium. Blank entries in this Table indicates that the gene was not differentially expressed

	T15		T30		T60	
Gene	Log2 FC	p value	Log2 FC	p value	Log2 FC	p value
*LSO1*	5.5	0.001	6.2	0.00008		
*SRX1*	5.5	0.015				
*HUG1*	4.7	0.001	5.2	0.00364	5.6	0.0321
*HBN1*	4.2	0.0179	2.9	0.0432		
*BTN2*	4.1	0.0171				
*ROQ1*	3.7	0.0225				
*YKL071W*	3.5	0.0385				
*RNR3*	3.4	0.00007	3.8	0.00857	2.9	0.0348
*MET17*	3.4	0.00275				
*HMX1*	3.3	0.00008	3.5	0.00352		
*YJL133C-A*			3.7	0.00324	3.5	0.00103
*FIT2*			3.4	0.00125	2.4	0.0105
*SPS100*			3.4	0.00818		
*TIS11*			3.3	0.0126		
*HSP82*			2.9	0.0426		
*YPR015C*					2.5	0.0209
*SPO24*					2.2	0.00982
*ANS1*					1.6	0.00068
*YLR053C*					1.2	0.0272
*CIS1*					1.2	0.000001
*THI12*					1.2	0.00576

**Table 2:** Differential expression of Iron Regulon Transcripts. Transcripts of genes identified as part of the iron regulon which were significantly (Log2 FC >1, *P* < 0.05) modulated as a function of laser irradiation time. Blank entries in this Table indicates that the gene was not differentially expressed. This list does not include any ESR genes

	T15		T30		T60	
Gene	Log2 FC	p value	Log2 FC	p value	Log2 FC	p value
*LSO1*	5.531574	0.00107	6.219368	8.04E-05		
*HMX1*	3.309266	8.09E-05	3.527614	0.00352		
*TIS11*	3.125413	0.00891	3.251401	0.0126		
*CCC2*	2.46325	0.00455	2.304157	0.0173		
*MRS4*	2.051588	0.0184				
*FRE3*	1.729197	0.0167	1.03597	0.0255		
*FIT2*	1.572728	0.0293	3.442218	0.00125	2.375446	0.0105
*ISU2*	1.537686	0.0169				
*FRE5*	1.450233	0.0323				
*FTH1*	1.305262	0.018				
*ARN2*	1.01569	0.021				
*FET4*	−1.10737	0.0264				

The transcriptional regulation of the iron regulon genes in UV-laser irradiated cells is likely in response to the generation of reactive oxygen species (ROS) which have the potential to cause intracellular damage to DNA, protein and lipids. ROS can result in decreased iron availability due to its oxidation (Matsuo *et al.* 2017) and many genes in the Aft1p/Aft2p-iron regulon are differentially expressed under oxidative stress (Castells-Roca *et al.* 2011; Castells-Roca *et al.* 2016). The iron regulon genes, including those identified in this study, encode proteins required for mobilization of iron, such as reductive and non-reductive uptake at the plasma membrane, reduction and transport from the vacuoles. The reductive pathway involves the conversion of ferric to ferrous ions, which can further contribute to oxidative stress by generating hydroxyl radicals from oxygen. The up-regulation at T15 and T30 of the transcript for Hmx1p, which regulates the expression of antioxidant genes (Collinson *et al.* 2011) in addition to regulating iron homeostasis, suggests a need for oxidant protection. *FIT2*, a cell wall mannoprotein transporter for siderophore-ferric iron was upregulated at T15, T30 and T60 suggesting intracellular ion scarcity. Under conditions of oxidative stress, gene expression patterns are altered to balance the essential need for iron homeostasis with minimization of the role of iron in the generation of hydroxyl radicals. We observed differential gene expression of iron regulon genes reflected in the response to UV-laser irradiation.

### Identification of highly modulated transcripts

**Figure 5:**
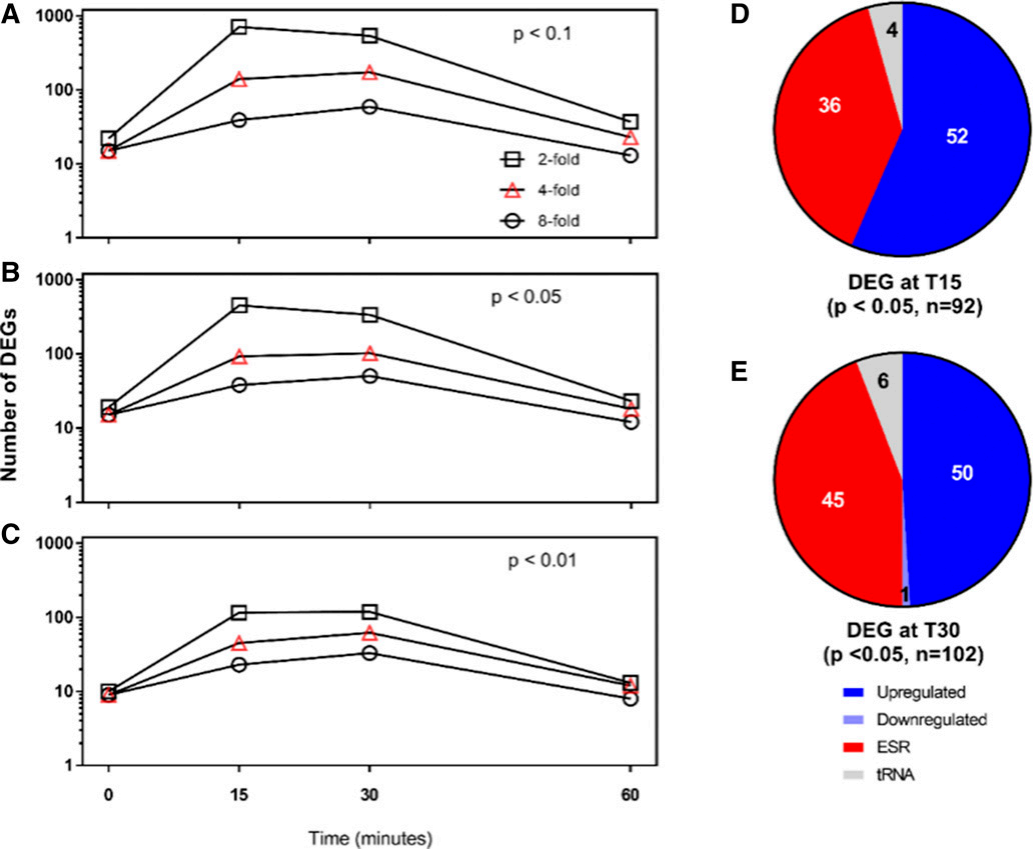
Differential transcript expression for control and laser treatment RPKM values at minimum twofold (□), fourfold (∆) or eightfold (○) change. Transcripts were sorted by p value (A) *P* < 0.1, (B) *P* < 0.05 and (C) *P* < 0.01. The transcripts differentially expressed (upregulated or downregulated), environmental stress response genes (ESR) or tRNA genes detected at T15 and T30 with *P* < 0.05 in panel B are characterized in panels (D) and (E), respectively. Figure 6. GO Slim annotation for cellular processes affected by laser irradiation at T15 (black, 73 transcripts) T30 (blue, 78 transcripts) for non-ESR transcripts with at least fourfold increase in expression at *P* < 0.05.

The conventional default values for identifying transcripts exhibiting differential expression is Log_2_ FC >1 (twofold change) at a p-value < 0.05. To identify transcripts which exhibited significantly higher levels of differential expression we plotted the number of differentially expressed genes at three different expression levels (≥twofold, ≥fourfold or ≥eightfold) as a function of time for three different p values (*P* < 0.1, *P* < 0.05 and *P* < 0.01). The number of genes expressing at least twofold change (*P* < 0.1) was the lowest at Time 0 and Time 60 (Figure 5A). Increasing the stringency (*P* < 0.05, *P* < 0.01; Figure 5B and 5C, respectively) decreased the absolute number of DEGs, but the greatest changes still occurred at T15 and T30. For this analysis, we elected to focus on genes differentially expressed at fourfold or higher, at a confidence interval of 95% at both T15 (Figure 5D) and T30 (Figure 5E) as a representative population for transcripts with a high degree of statistically significant differential expression. Using these stringent criteria, 92 and 102 transcripts were identified at T15 and T30, respectively (listed in Table S4). Because laser irradiation should serve as a stress condition, this pool of differentially expressed genes was assessed for the presence of ESR genes. Of the 92 genes identified at T15, 36 were upregulated ESR genes; of the remaining population 52 genes were upregulated and 4 tRNA genes were downregulated. The distribution of DEGs at T30 was similar to that at T15, with 45 ESR genes upregulated, 6 tRNA genes (1 upregulated, 5 downregulated) and 51 genes (50 upregulated, 1 downregulated) were differentially expressed in response to laser irradiation.

**Figure 6:**
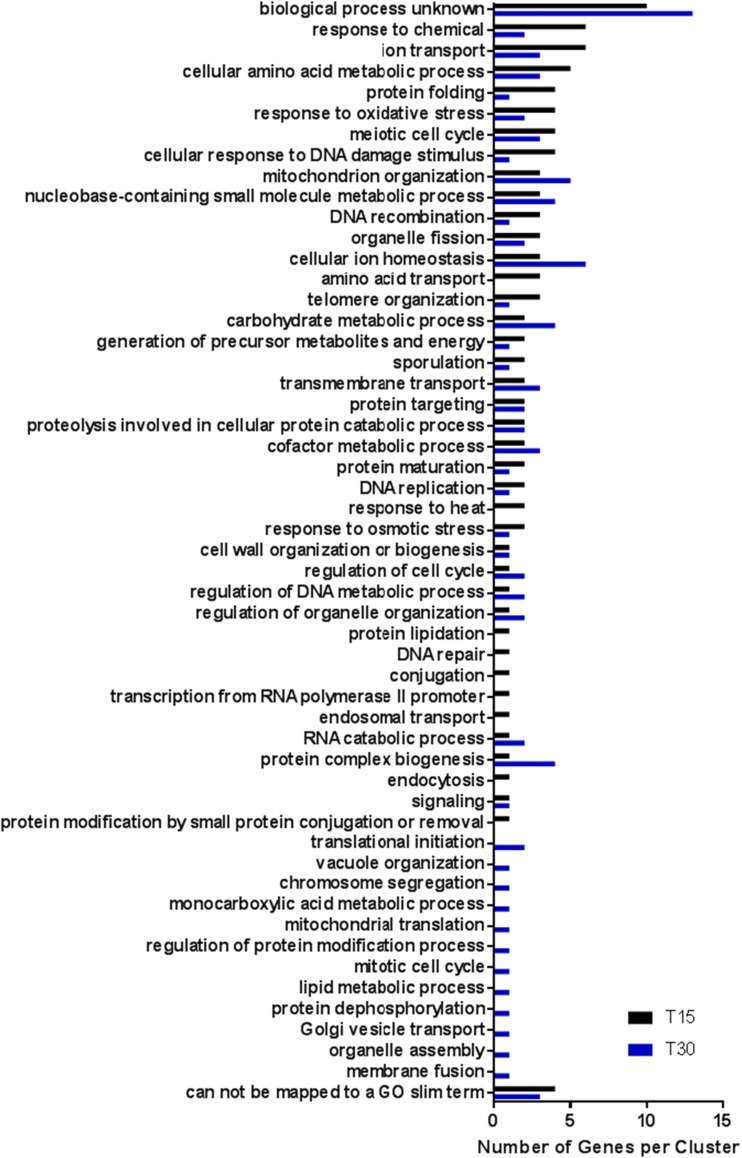
GO Slim annotation for cellular processes affected by laser irradiation at T15 (black, 73 transcripts) T30 (blue, 78 transcripts) for non-ESR transcripts with at least fourfold increase in expression at *P* < 0.05.

The non-ESR genes differentially expressed in response to irradiation were grouped by cellular process using GO Slim Mapper at T15 and T30 (Figure 6). The largest cluster mapped to the category “biological process unknown” for both T15 (10/52 genes) and T30 (13/51 genes). At the time of this analysis, a function has not been assigned to these genes. There were also genes which could not be associated with a GO Slim term at both T15 (4/52 genes) and T30 (3/51 genes). Among these were a component of the iron regulon *LSO1* (An *et al.* 2015) previously mentioned as a highly differentially expressed transcript at both T15 and T30 (Table 1 and Table 2). Other non-assigned genes at T15 were annotated in the Saccharomyces Genome Database (Cherry *et al.* 2012) as encoding an aryl-alcohol dehydrogenase (*AAD4*) and a flavin mononucleotide (*OYE3*), both involved in oxidative stress response, and a glutathione S-transferase (*GTT2*) which is upregulated in response to DNA replication stress. At T30, unassigned genes were *FIT2*, part of the iron regulon (Table 1 and Table 2) and *STF1* which encodes a protein involved in the regulation of the mitochondrial F1F0-ATP synthase upregulated in response to DNA replication stress.

Discounting the transcripts which could not be mapped to a GO Slim term or for which the biological function is unknown at T15, the remaining 38 differentially expressed transcripts were assigned to 39 cellular process categories. At T30, the 35 differentially expressed genes which did not include genes which could not be mapped or had no known biological function were assigned to 42 different processes (Figure 6 and Table S4). At T15, processes related to biological transport (*i.e.*, ion transport, transmembrane transport, cellular ion homeostasis, amino acid transport) are represented. Reflecting the results of the hierarchical clustering analysis (Figure 4B), transcripts for amino acid transport genes (*BTN2*, *MUP3*, *YCT1*) involved in arginine (*BTN2*), methionine (*MUP3*) and cysteine (*YCT1*) transport were highly differentially expressed (*P* < 0.05, ≥ fourfold change) only at T15, suggesting an early upregulation, which is not detected at this same stringency at T30. Interrogation of the transcriptome at lower stringency (*P* < 0.1, ≥ twofold change) identified the four amino acid transporters identified above, as well as general amino acid permease *GAP1* and the *PUT4* proline transporter. A similar interrogation at reduced stringency for the GO process “amino acid transport” at T30 identified only *AGP3*, a low-affinity amino acid permease which may function to transport amino acids for use as a nitrogen source in nitrogen-limiting conditions. None of the other amino acid transport genes differentially expressed at T15 were detected at T30 further underscoring the transient nature of differential gene expression when comparing these two time points. These transport responses may indicate a cellular mobilization of external resources to respond to the laser insult. Amino acid would be required for rapid protein synthesis rather than to await intracellular biosynthesis of these building blocks. The regulation of this specific set of amino acid transporters is interesting and warrants further exploration.

At T30 the most highly represented processes for the 35 differentially expressed genes described above included cellular metabolism (carbohydrate metabolic process, protein complex biogenesis, cellular amino acid metabolism), ionic homeostasis (cellular ion homeostasis, ion transport, transmembrane transport), mitochondrion organization, and sporulation. These changes may indicate a complex cellular response to the laser insult in comparison to the more immediate responses at T15. Mobilization of energy sources requiring mitochondrial function, carbohydrate metabolism, protein turnover and biosynthesis would be necessary. The initiation a sporulation response might indicate a survival mechanism as well. These results indicate that both mitochondrial and cellular components are the targets of oxidative damage due to long-wavelength UV radiation.

UV-irradiation would be expected to result in damage to DNA, protein and other macromolecules. Interestingly, only 4 genes (*ECO1*, *RAD53*, *FRA1*, and *RFA2*) out of 63 in the Saccharomyces Genome Database (SGD, www.yeastgenome.org) annotated with the Gene Ontology (GO) term “DNA Repair” were up-regulated at the stringent fourfold, 95% confidence level at T15. At T30, *RFA1*, *RFA2*, and *SOH1* were the only DNA Repair transcripts up-regulated (Table S1, DNA Repair tab). That more genes involved in DNA repair were not highly up-regulated was likely due to the long-wavelength UV (355nm) and the short irradiation time used in our experiments; short-to-mid wavelength UV (250-300nm) is primarily responsible for UV damage to DNA (Rastogi *et al.* 2010; Besaratinia *et al.* 2011).

There was a representation of genes annotated in SGD as involved in the phenotype “UV Resistance” primarily documenting genes involved in UV repair after exposure to shorter wavelength UV 0f 300 nm or less (https://www.yeastgenome.org/observable/APO:0000085) with 27 and 22 genes out of 322 total genes annotated to this term up-regulated at T15 and T30, respectively, with many of these genes overlapping at the two time intervals (Table S1, UV Resistance Tab) suggesting a prolonged response. Notably, the transcription factor *YAP1*, which plays a central role in response to oxidative stress tolerance (Morano *et al.* 2012) was upregulated. *YAP 1*, a basic leucine zipper (bZIP) transcription factor is a non-essential gene; a null mutant showed increased mutation frequency and decreased resistance to short-wave UV radiation and oxidizing and reducing agents while overexpression conferred resistance to these agents (Temple *et al.* 2005; Toone and Jones 1998). Our results indicate that *YAP1* is also involved in conferring resistance to long-wavelength UV. In addition, 6 different RAD (RADiation sensitive) genes (*RAD16*, *RAD51*, *RAD52*, *RAD53*, *RAD54*, *RAD 59*, see Table S1, UV-resistance tab) which are involved in nucleotide excision repair in response to DNA damage by UV light (Aboussekhra and Wood 1994) were also upregulated as was *HUG1*, a gene previously shown to be up-regulated by short-wavelength UV(Wade *et al.* 2009), suggesting that if there is some level of DNA damage, it is efficiently repaired by the genes activated in response to oxidative stress and does not result in any compromise of cell growth.

At T15 there was an up-regulation of 15 of the 31 transcripts from genes annotated as “Proteosome Storage Granule” in SGD (Table S1, Proteasome tab). This number of up-regulated genes related to proteasome function increased to 21 genes at T30. These data provide evidence of protein turnover being a major cellular response to long-wavelength UV-irradiation. The corresponding ubiquitin ligase genes, which function in targeting of proteins for degradation, were represented by transcripts of *CDC48*, *NPL4*, and *UFD1* at T15 and by *UFD1* only at T30 (Table S1, Ubiquitin Ligase tab).

### Differential expression of tRNA genes

A recent report (Torrent *et al.* 2018) indicates that the tRNA pool in yeast changes in response to a diverse collection of stress conditions in order to enhance selective translation of stress-induced transcripts. In our study, over the 60-minute sampling time, seventeen different transcripts encoding tRNA genes were differentially expressed (*P* < 0.05) in response to laser irradiation (Table 3). In contrast to other highly regulated genes, most of the tRNA transcripts exhibited primarily reduced expression. Downregulation occurred at T0 for the *EMT2* (Log_2_ FC = -3.0) a methionine tRNA involved in adding methionine residues to the nascent peptide chain. Interestingly, the transcript for *IMT4* which encodes a methionine initiator tRNA was up-regulated (Log_2_ FC = 3.0) at T30 suggesting an increase in protein synthesis. The largest change in expression occurred at T15 for a leucine tRNA (Log2 FC = - 4) which was downregulated by 16-fold. Other tRNA genes were also regulated, but at levels below the threshold applied for significance (Table S1). These data indicate these tRNAs were regulated by laser-induced damage. We surmise that these tRNA transcripts were captured in the RNAseq analysis due to polyA tails known to be present in some tRNA transcripts (Düvel *et al.* 2003; Torrent *et al.* 2018) or possibly added during processing as a means to down-regulate tRNAs to control translation (Wilusz 2015; Phizicky and Hopper 2010). It is probable that only a small set of tRNA down-regulation would be needed to stop protein synthesis entirely to repair cellular damage in preparation for re-initiating cellular growth after repairs had been done.

**Table 3:** Transcripts for tRNA genes differentially expressed at T0, T15, T30, and T60

Control v. Laser T0				
Name	tRNA Gene	P value	Log2 Fold Change	
*tQ[UUG]E1*	Glutamine (thiolation of uridine at wobble position)	0.00272	3.0	up
*tK[UUU]G2*	Lysine (thiolation of uridine at wobble position)	5.21E-06	2.3	up
*EMT2*	Met (elongation)	0.0152	3.0	down
Control v. Laser T15				
Name	tRNA Gene	P value	Log2 Fold Change	
*tL[CAA]G1*	Leucine	1.35E-05	4.0	down
*tR[ACG)J*	Arginine (one of 6 nuclear tRNA genes containing the tDNA-anticodon ACG (converted to ICG in the mature tRNA), decodes CGU, CGC, and probably CGA codons into arginine, one of 19 nuclear tRNAs for arginine	0.0062	3.8	down
*tD[GUC]J2*	Aspartate	1.12E-03	2.7	down
*tV[AAC]E1*	Valine	4.99E-04	2.4	down
Control v. Laser T30				
Name	tRNA Gene	P value	Log2 Fold change	
*IMT4*	Methionine (initiator)	1.58E-03	3.0	up
*tN[GUU]L*	Asparagine	6.54E-04	2.5	down
*tR[UCU]M2*	Arginine (nuclear)	1.77E-03	2.4	down
*tN[GUU]N1*	Asparagine	1.79E-03	2.3	down
*tH[GUG]E1*	Histidine	5.64E-05	2.1	down
*tK[CUU]C*	Lysine (mito with Msk1p)	5.68E-05	2.1	down
Control v. Laser T60				
Name	tRNA Gene	P value	Log2 Fold change	
*tK[UUU]G1*	Lysine (thiolation of uridine at wobble position)	0.000739	2.2	up
*tA[AGC]M2*	Alanine (one of 11 nuclear tRNA genes containing the tDNA-anticodon AGC (converted to IGC in the mature tRNA), decodes GCU and GCC codons into alanine, one of 16 nuclear tRNAs for alanine	8.18E-07	2.0	up
*tH[GUG]H*	Histidine	0.00011	2.7	down
*tG[GCC]O1*	Glycine	0.00205	2.4	down

## Conclusion

Exposure to UV irradiation as delivered in this study (355 nm, 30 sec, 55 J) allowed for continued cell viability, but resulted in rapid, transient changes in gene expression assayed at T15 and T30 min post-transfer of the irradiated cells into fresh growth medium which returned to baseline levels by T60. UV-irradiation is a source of cellular stress; we did observe differential expression of a subset of the ESR genes (Gasch *et al.* 2000). However, upon filtering out the ESR genes, as well as setting a stringent selection criterion to identify transcripts with at least a fourfold change in expression, we identified a population of transcripts (56 at T15 and 57 at T30) that we ascribed to the laser-irradiated transcriptome.

The cell responded to long-wavelength UV irradiation with transcriptional upregulation of genes annotated as responding to UV resistance and oxidative stress. Furthermore, consistent with a cellular response to repair cellular damage and to prepare for cellular growth upon introduction into fresh medium, we noted increased transcripts for amino acid mobilization and protein turnover utilizing proteasome components, proteases, and peptidases. The down-regulation of tRNA genes also points to protein translation as a key process in the cell’s coping with UV irradiation-induced damage. That DNA repair was not a large factor in response to the UV laser used in this experiment is also consistent with the know targets of long-wavelength UV as opposed to shorter-wavelength UV-induced DNA damage. In addition, it is known that the transcriptional response of *S. cerevisiae* to short-wavelength UV radiation that damages DNA may not directly identify genes that protect against UV radiation (Birrell *et al.* 2002). The yeast cell demonstrates a notable ability to respond to damaging UV irradiation over a short time interval by returning the normal transcriptional responses by 60 min post-irradiation.

Long-wavelength UV radiation has been used to a much lesser extent in biological studies in comparison to the use of short-wavelength UV. Experiments using long-wavelength UV have concentrated mainly to crosslink psoralen to DNA as this compound is used to treat skin diseases. Some studies have measured transcriptional responses to long-wavelength UV in keratinocytes (Luo *et al.* 2017; Kraemer *et al.* 2013; Zhao *et al.* 2013) and in melanocytes (Zhao *et al.* 2017). In yeast, long-wavelength UV and psoralen has been used as a model to study DNA damage repair mechanisms (Cruz *et al.* 2012).

The results of these experiments demonstrate that a short burst of high-energy, long-wavelength UV did not substantially impact cell viability. Furthermore, the transient nature of the transcriptional response and the modest number of genes whose transcription was regulated under the conditions of these experiments indicates that long-wavelength UV irradiation of yeast cells for a short time period would not impact proteomic studies unless the expression of a particular target protein or its interactors were affected. There should be no additional *in vivo* cross-linking of cellular proteins that were not already in close physical proximity at the initiation of the laser-induced cross-linking.
